# Circular RNA Circ_0000677 promotes cell proliferation by regulating microRNA-106b-5p/CCND1 in non-small cell lung cancer

**DOI:** 10.1080/21655979.2021.1965697

**Published:** 2021-09-14

**Authors:** Xin Hu, Ping Wang, Chen Qu, Haibo Zhang, Liang Li

**Affiliations:** a Department Of Internal Medicine, Nantong Maternity And Child Health Hospital, Nantong, China; b Department Of Geriatrics, The Second Affiliated Hospital of Nanjing Medical University, Nanjing, China; c Department Of Emergency Medicine, Shanghai Seventh People’s Hospital, Shanghai, China

**Keywords:** Circ_0000677, miR-106b-5p, ccnd1, proliferation, non-small cell lung cancer

## Abstract

Recently, circular RNAs (circRNAs) have become an intense focus of research and large numbers of circRNAs have been identified, awaiting functional elucidation. Thus, the present study aims to examine the regulation of circRNAs and its molecular mechanism in lung cancer growth. Here, we show that circular RNA circ_0000677 was overexpressed and correlated with poor prognosis in non‐small cell lung cancer (NSCLC) patients. Functionally, circ_0000677 knockdown markedly inhibited proliferation of NSCLC cells by observing of immunofluorescence staining of Ki67, clone formation assay, and xenograft experiments. In mechanism, circ_0000677 acted as a sponge of microRNA-106b and further regulated CCDND1 gene expression in NSCLC cells by dual luciferase activity assay and their expression examination. Taken together, these findings suggest a role for circ_0000677/miR-106b/CCND1 regulation axis in promoting NSCLC growth and progression.

## Introduction

Lung cancer is the most common cancer, and the leading cause of death around the world [1]. The morbidity and mortality of lung cancer have continued increasing every year, posing a great threat to health worldwide [2]. Histopathologically, lung cancer can be divided into small cell lung cancer (SCLC) and non-small cell lung cancer (NSCLC), of which NSCLC accounts for approximately 80% or more of all total pulmonary malignancies [3]. Despite of the advancements in therapeutic measures, the overall survival remains quite poor [4]. Therefore, understanding the mechanisms underlying NSCLC development and progression is essential for efficient therapies for NSCLC patients and achieving a better prognosis.

Circular RNAs (circRNAs), a type of recently discovered noncoding RNA, are widely found in biological systems [5]. CircRNAs are covalently closed loop RNAs, which form a stable structure and can be protected from degradation of RNase enzymes [6]. Based on well understanding of their molecular features, regulation of circRNAs on multiple biological processes has been receiving increased research attention. Previous studies reported that circRNAs are closely related to progression of various tumors, including lung cancer [7–10]. For example, the well-known circRNA CDR1 (ciRS-7), which functions as a miRNA inhibitor, has been suggested to play essential roles in tumor growth and metastasis by considerable experiments [11–13]. In addition, it has been well elucidated that circRNAs can function as early detective biomarkers and participate in cancer cell stemness regulation and drug resistance [14–16]. Yet the functions and regulation mechanisms of these special loop RNAs in tumors, especially in NSCLC, are only partially understood.

Circular RNA circ_0000677 (Alias: circ_001569) is a newly discovered circRNA of 1776 bp and located on chromosome 16q13.1. Circ_0000677 has been found to be overexpressed in multiple types of tumors [17–20]. It has been verified that circ_0000677 is an effective biomarker of certain tumors and often portends a poor prognosis in patients [21–23]. In mechanism, circ_0000677 is considered to be involved in regulation of cancer cell proliferation, migration, invasion and drug resistance [24,25]. Thus, we hypothesize that circ_0000677 and its regulation of downstream targets might play a role in NSCLC development. In the present study, we aimed to evaluate the expression levels of circ_0000677 in NSCLC tissues and explore the molecular mechanisms underlying NSCLC progression. The discovery of circ_0000677 and its downstream signaling axis regulating NSCLC cell growth may provide new insights to understand mechanisms underlying NSCLC development and thus yield new therapeutic strategies.

## Methods

### Patient samples

The human NSCLC specimens and paired normal adjacent tissues were obtained from 35 patients underwent a surgical procedure at the Nantong Maternity and Child Health Hospital andThe Second Affiliated Hospital of Nanjing Medical Universityfrom August 2019 to March 2021. After surgery, all specimens were immediately frozen in liquid nitrogen and stored at −80°C. All the patients provided written consent, and the Ethics Committee from Nantong Maternity and Child Health Hospital (No. Y2017096) approved all aspects of this study.

### Cell culture and plasmid transfection

The human NSCLC lines (NCI-H358, NCI-H1650, NCI-H1568, HCC827, and A549, NCI-H1299) were purchased from Shanghai Institute of Cell Biology, Chinese Academy of Sciences (Shanghai, China). HEK-293 T cell was maintained in DMEM medium (Gibco, USA) and other cells were cultured in RPMI-1640 (Gibco, USA), supplemented with 10% fetal bovine serum (Gibco, USA), and 1% penicillin and streptomycin (Gibco, USA). The cultured cells were maintained at 37°C in a humidified 5% CO_2_ incubator. Small interfering RNAs (siRNAs) transfection and plasmid were conducted as described [26]. When cells reached 40–60% confluence, they were transfected for 48–72 h with siRNAs (circ-in, 5′-GCATCGTGCAGGACTGGAA-3′) targeted to circ_0000677 (constructed by Sangon, Shanghai) at a 50-nM concentration. A scrambled siRNA was utilized as a negative control. Transfection was performed with the lipofectamine 3000 according to the manufacturer’s instructions. To establish stable cell lines overexpressing CCND1, cells were transfected with a pCMV6-CCND1 plasmid DNA (Origene) or the empty plasmid served as the negative control. Human CCND1 cDNA cloned in pCMV6-XL5 was purchased from ORIGENE.

### RNA extraction and quantitative real-time polymerase reaction (qRT-PCR)

Total RNA was extracted from cultured cells and tissues with TRIzol reagent (Takara, Japan), according to the manufacturer’s protocol. Reverse transcription of miRNA was then conducted using PrimeScript RT Reagent Kit (Takara, Japan) with stem-loop primers. For reverse transcription of mRNA and circRNA, PrimeScript RT Master Mix (Takara, Japan) with random primers was used. The real-time PCR was performed on an Applied Biosystems 7500 Sequence Detection System (Applied Biosystem, USA) with TB Green Premix Ex Taq II (Takara, Japan) as described [27]. GAPDH and U6 were used as internal references for quantification. Relative expressions of miRNA, circRNA and mRNA were calculated using the formula 2 – ^ΔΔCT^.

### Western blot

Western blot analysis was performed as described [28]. Total protein of NCI-H1299 cells was extracted with RIPA lysis buffer (Beyotime, China) and separated by 10% SDS-PAGE, then transferred onto a polyvinylidene fluoride (PVDF) membrane (Millipore, USA). The membranes were blocked with 5% skim milk powder and incubated with primary antibody anti-CCND (1:10,000, Abcam, USA) and anti-GAPDH (1:5000, Cell Signaling Technology, USA) at 4°C overnight. After incubation with the ensuing secondary antibodies (1:5000, Cell Signaling Technology, USA) at room temperature for 2 h, the bands were finally visualized by ECL chemiluminescent reagent (Millipore, USA).

### Luciferase reporter assay

For the luciferase reporter assays, the wide type (WT) sequences of circ_000067 and CCND1, and their corresponding mutation (MT) were synthesized and inserted into luciferase reporter vector GP-miRGLO (Genepharma, China), as detailed in the ‘Results’ section. All these WT- or MT-plasmids were co-transfected with equal amounts of mim-miR-106b-5p, or mim-miR-control to HEK293T cells using Lipofectamine 3000 (Invitrogen), respectively. Twenty-four hours after transfection, the cells were assayed using a Dual Luciferase Assay kit (Promega, USA) as described [29].

### Xenograft experiments

For the tumorigenicity assay, 5-week-old male BALB/c nude mice were purchased from the Model Animal Research Center at hospital. Xenograft experiment was performed as described [30]. Total of 5 × 10^6^ Fluc-labeled NCI-H1299 cells transfected with si-circ_0000677 or control vector were subcutaneously injected into the left hinder leg, respectively. Luciferase activity, i.e. the growth of tumors in vivo, was measured by bioluminescence imaging after mice received D-luciferin (Promega, USA) in PBS at a final concentration of 0.15 mg/ml. Machines for bioluminescence imaging used in this study was IVIS Lumina Series III (PerkinElmer, USA). The study has obtained permission from the Ethics Committee from Yancheng Third People’s Hospital, met the standards set out in the NC3Rs primate’s guidelines and followed best practice procedures.

### Immunofluorescence (IF) staining

IF analysis was performed as described [31]. Cells grown on cover-glasses were fixed in 4% paraformaldehyde, permeabilized with 0.3% Triton X-100, and blocked with 5% bovine serum. Then the cells were incubated with antibodies against Ki67 (1:500, Abcam, USA) overnight at 4°C, followed by Alexa Fluor 594-conjugated secondary antibodies (1:500, Abcam, USA). Nuclei were dyed by DAPI and images were observed under a fluorescence microscope (Leica, Germany).

### Colony formation assay

Colony formation assay was performed as described [32,33]. Cells were plated at a density of 1000 cells per well in 6-well plates and then cultured at incubator for 2 weeks. The prepared cells were then fixed with 4% paraformaldehyde and stained with a 0.5% crystal violet solution for 15 min at room temperature. The colonies containing more than 50 cells were counted and analyzed.

### Statistical analysis

All statistical analyses were performed by GraphPad Prism 8.0. Data are presented as mean±s.e.m. Student’s *t* test and one-way ANOVA were used for statistical analysis. Survival was analyzed by Kaplan-Meier survival curve and correlation was analyzed using Pearson correlation test. P < 0.001 was considered statistically significant.

## Results

### Upregulation of circ_0000677 in non-small cell lung cancer

Circ_0000677 has been considered to be associated with tumorigenesis and prognosis in multiple tumor [17–19]. We hypothesize that circ_0000677 is crucial for regulating growth of NSCLC cell and might be a potential therapeutic target. In the present study, we found that high expression of circ_0000677 and its cell cycle regulation via functioning as miRNA sponge caused malignant proliferation during the progression of NSCLC.

**Figure 1.**
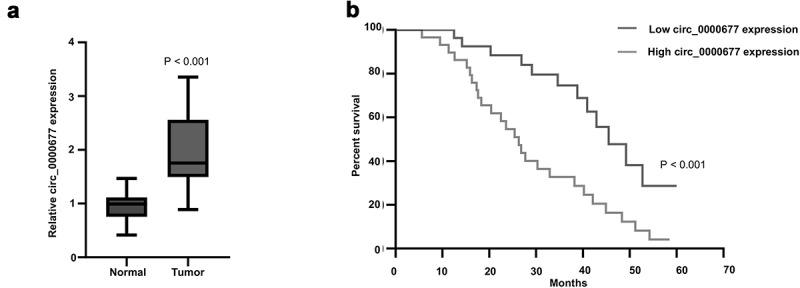
High expression level of circ_0000677 predicts unfavorable prognosis in NSCLC patients

In order to reveal the expression profiles of circ_0000677 in non-small cell lung cancer, 30 paired NSCLC tumor tissues and their corresponding adjacent non-cancerous tissues were detected. PCR analysis demonstrated that circ_0000677 was significantly higher expressed in NSCLC tissues as compared to normal lung tissue (Figure 1a). Considering that circ_0000677 was closely related to cancer progression, we decided to determine whether the expression levels of circ_0000677 in NSCLC correlate with the prognosis. 60 NSCLC patients were divided into high circ_0000677 expression group and low circ_0000677 expression group, based on the median value of ISH scores for circ_0000677 expression in tumor tissues. Kaplan-Meier curve analysis showed that NSCLC patients in high circ_0000677 expression group had worse overall survival than those in low circ_0000677 expression group (Figure 1b). Overexpressed Circ_0000677 contributed to the worse prognosis of NSCLC patients based on this 60-month investigation.

(a) Expression of circ_0000677 in 30 paired NSCLC tumor tissues and their corresponding adjacent noncancerous tissues. The results are represented as box plots with the median indicated by the horizontal line. (b) The survival curve showing the prognostic significance of circ_0000677 patients with NSCLC. 60 NSCLC patients were divided into high circ_0000677 expression group (30 cases) and low circ_0000677 expression group (30 cases), based on the median value of ISH scores for circ_0000677 expression during a total of 60 months observation time.

### Circ_0000677 promotes the proliferation of lung cancer cells

**Figure 2.**
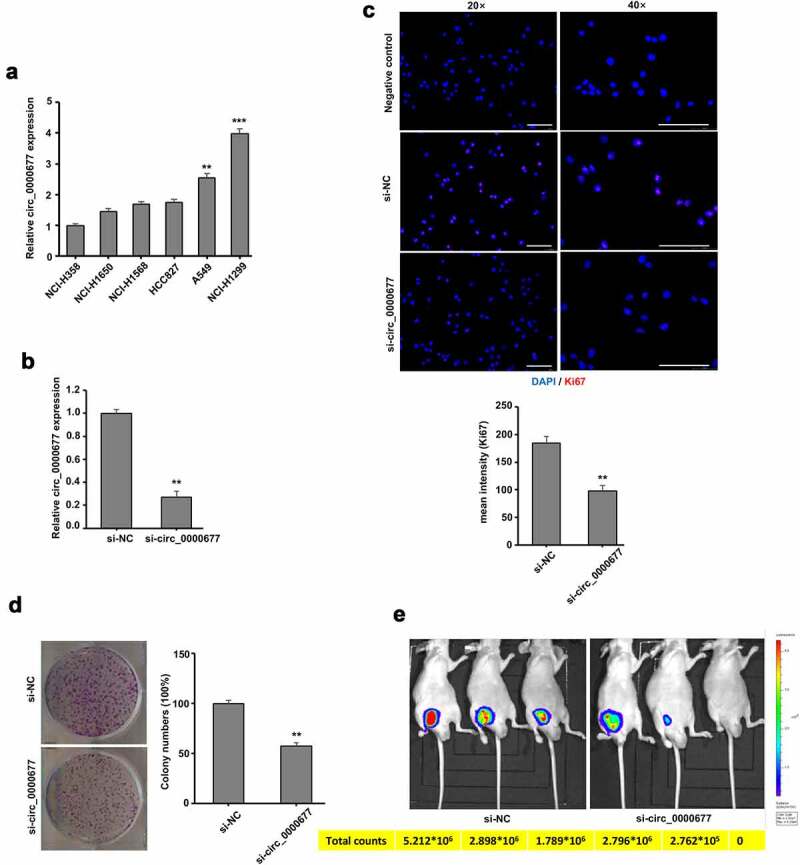
Circ_0000677 drives NSCLC cell proliferation *in vitro* and *in vivo.*

To further investigate the role of circ_0000677 in the proliferation in NSCLC cells, we examined circ_0000677 expression cross a panel of NSCLC cell lines. PCR analysis showed that circ_0000677 expression in A549 and NCI-H1299 was significantly higher than in other cell lines (NCI-H358, NCI-H1650, NCI-H1568 and HCC827) (Figure 2a). Thus, NSCLC cell line NCI-H1299 was picked and RNAi vector against circ_0000677 was constructed. The results showed that circ_0000677 was effectively knocked down in NCI-H1299 after RNAi vector transfection (Figure 2b). Immunofluorescence staining of Ki67 demonstrated that knockdown of circ_0000677 in NCI-H1299 cells significantly increased the percentages of Ki67-positive cells, which indicated elevated proliferation capacity of NSCLC cells (Figure 2c). Similarly, clone formation assay showed knockdown of circ_0000677 inhibited the clonal formation ability in NCI-H1299 cells (Figure 2d). To further examine the effects of circ_0000677 on tumor growth *in vivo*, NCI-H1299 cells were subcutaneously inoculated in BALB/c nude mice. After 4 weeks, the fluorescence image confirmed that knockdown of circ_0000677 attenuated the *in vivo* tumorigenic capacity of NCI-H1299 cells (Figure 2e). These results suggested circ_0000677 promoted the ability of NSCLC cell growth.

(a) Relative expression of circ_0000677 in six NSCLC cell lines measured by qRT-PCR. (b) RT-PCR analysis of relative circ_0000677 expression in NCI-H1299 cells with RNAi-mediated gene knock-down. (c) Immunofluorescence labeling of cell proliferation marker Ki67 in NCI-H1299 cells. The percentages of Ki67-positive cells after knockdown of circ_0000677 were detected (scale bar: 100 μm). (d) Representative image (left panel) and statistical plots (right panel) of the effect of circ_0000677 on clone formation capability of NSCLC cells NCI-H1299. (e) The fluorescence image showing *in vivo* characterization of the tumorigenic property of NSCLC cells in BALB/c nude mice after circ_0000677 knockdown. The total observation time for this treatment was 4 weeks.

### Circ_0000677 acts as the sponge of miR-106b

**Figure 3.**
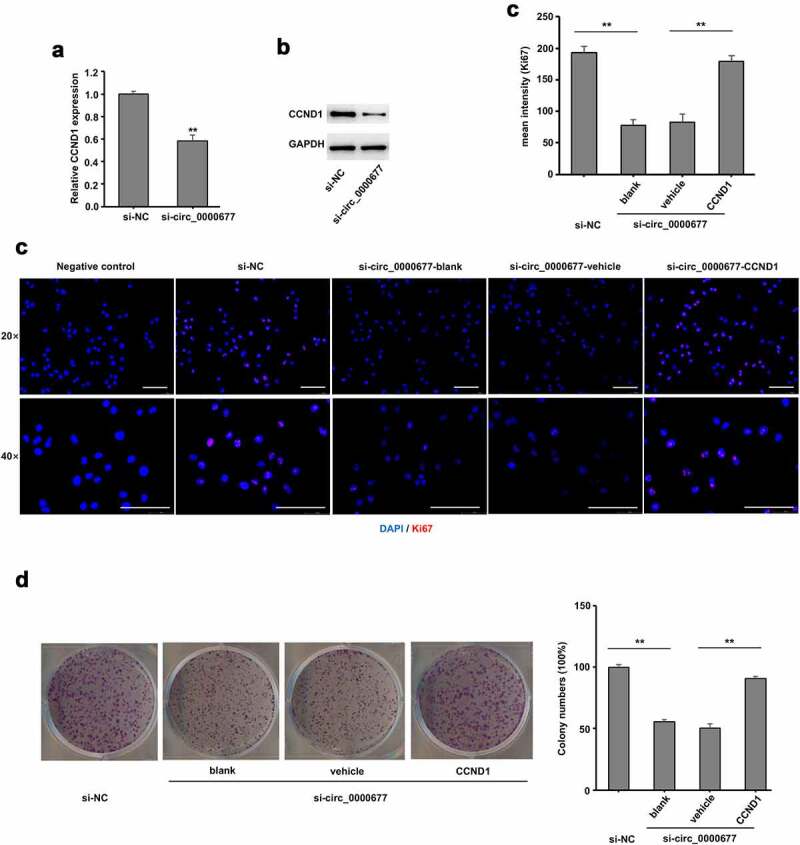
Circ_0000677 acts as a sponge of miR-106b

To understand the molecular mechanism by which circ_0000677 contributed to NSCLC cell proliferation, potential relationships between circ_0000677 and its target microRNA (miRNA) were predicted using TargetScan and miRanda database. The results showed that miR-106b might be targeted by circ_0000677, whose 3′-UTR region possesses a putative binding site for miR-106b (Figure 3a). Base on this, wild type (WT) and mutant type (MT) circ_0000677 3′-UTR luciferase reporter systems were designed (Figure 3a). Luciferase reporter assay showed that miR-106b-5p mimic transfection in 293 T cells led to decreased luciferase activity of wild-type circ_0000677 reporter, with no significant change in mutant type reporter (Figure 3b). Considering circ_0000677 could act as the sponge of miR-106b, qRT-PCR analysis showed that circ_0000677 knockdown unambiguously increased miR-106b expression in NSCLC cell line NCI-H1299 (Figure 3c). Further Pearson correlation analysis of tumor tissues from 35 NSCLC patients showed a markedly inverse correlation between circ_0000677 and miR-106b expression (Figure 3d). Taken together, these results suggested that circ_0000677 could function as a sponge of miR-106b in NSCLC.

(a) Schematic representation of the wild-type (WT) and mutant-type (MT) circ_00006773′-UTR luciferase reporter system, of which circ_0000677-WT 3′-UTR possesses a putative binding site for miR-106b-5p.B. 3′-UTR luciferase activity of WT and MT circ_0000677 in 293 T cells after miR-106b-5p mimic transfection. (c) RT-PCR analysis of relative miR-106b expression in NCI-H1299 cells with circ_0000677 knockdown. (d) Expression relationship between circ_0000677 and miR-106b in 35 NSCLC patients.

### CCND1 was negatively regulated by miR-106b

**Figure 4.**
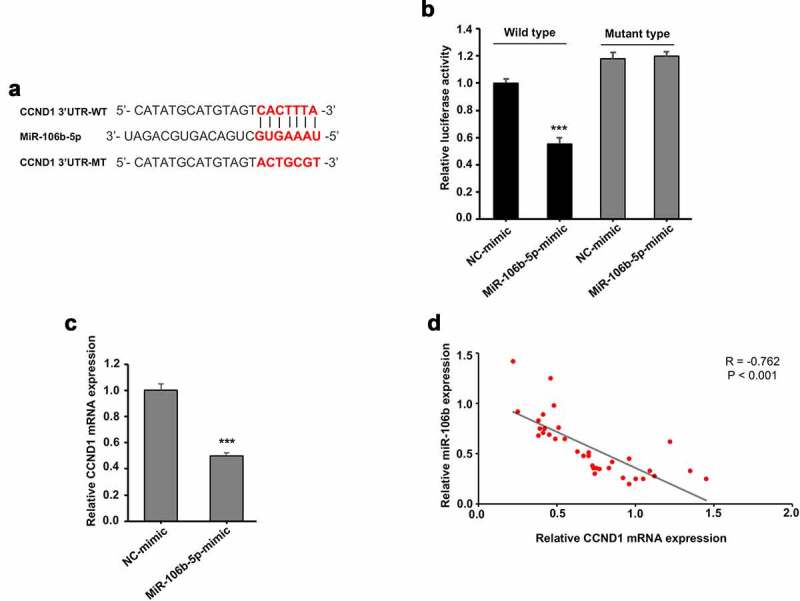
CCND1 was negatively regulated by miR-106b

In order to verify detailed molecular regulation of circ_0000677/miR-106b axis, target genes of miR-106b were searched for. We identified that CCND1 (cyclin D1) might be targeted by miR-106b, whose 3′-UTR region possesses a potential binding site for miR-106b (Figure 4a). Base on this, wild type (WT) and mutant type (MT) CCND1 3′-UTR luciferase reporter systems were designed (Figure 4a). Luciferase reporter assay showed that miR-106b-5p mimic transfection in 293 T cells led to decreased luciferase activity of wild type CCND1 3′-UTR reporter, with no significant change in mutant type reporter (Figure 4b). When miR-106b-5p mimic was transfected in NSCLC cell NCI-H1299, qRT-PCR analysis revealed markedly reduced mRNA expression of CCND (Figure 4c). Likewise, Pearson correlation analysis of tumor tissues from 35 NSCLC patients showed a markedly negative correlation between CCND and miR-106b (Figure 4d). Taken together, these results suggest that CCND1 was negatively regulated by miR-106b.

(a) Schematic representation of the wild-type (WT) and mutant-type (MT) CCND1 3′-UTR luciferase reporter system, of which CCND1-WT 3′-UTR possesses a putative binding site for miR-106b-5p.B. 3′-UTR luciferase activity of WT and MT CCND1 in 293 T cells after miR-106b-5p mimic transfection. (c) RT-PCR analysis showing relative mRNA expression of CCND1 in NCI-H1299 cells with miR-106b-5p mimic transfection. (d) Expression relationship between CCND1 and miR-106b in 35 NSCLC patients.

### Circ_0000677/miR-106b/CCND1 axis in promoting NSCLC proliferation

**Figure 5.**
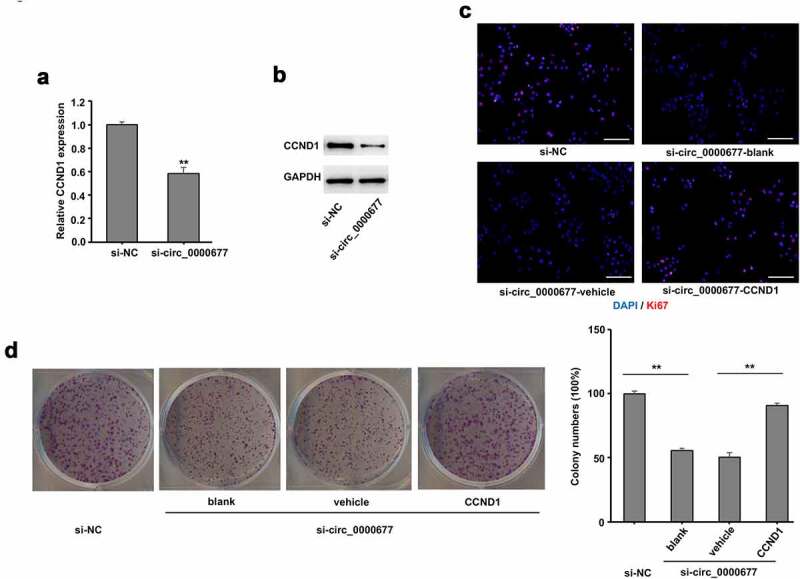
Upregulation of CCND1 expression reversed the inhibition effect of circ_0000677 knockdown on proliferation of NSCLC cells

To further confirm CCND1 plays a role in CCND1/ miR-106b regulation, circ_0000677 knock down was conducted in NSCLC cell line NCI-H1299, and qRT-PCR analysis revealed an unambiguously reduced mRNA expression level of CCND1 (Figure 5a). Likewise, protein expression level of CCND1 was markedly decreased in NCI-H1299 cells with circ_0000677 knocked down, revealed by Western blot analysis (Figure 5b). Immunofluorescence staining of Ki67 showed that CCND1 restoration markedly increased the percentages of Ki67-positive cells in NCI-H1299 with circ_0000677 knocked-down, which indicated an elevated proliferation capacity of NSCLC cells (Figure 5c). Likewise, clone formation assay demonstrated that knockdown of circ_0000677 inhibited the clonal formation ability in NCI-H1299 cells, while CCND1 restoration rescued the inhibition effect of circ_0000677 knockdown on NSCLC cell proliferation (Figure 5d). These results together suggested that circ_0000677/miR-106b/CCND1 axis regulated proliferation of NSCLC cells.

(a) RT-PCR analysis showing relative mRNA expression of CCND1 in NSCLC cell line NCI-H1299 with circ_0000677 knockdown. (b) Western blot analysis of CCND1 expression in NCI-H1299 cells with circ_0000677 knockdown. (c) Immunofluorescence labeling of cell proliferation marker Ki67 in NCI-H1299 cells. The percentages of Ki67-positive cells in NCI-H1299 cells were detected after circ_0000677 knockdown and CCND1 restoration (scale bar: 100 μm). (d) Representative image (left panel) and statistical plots (right panel) of the effect of circ_0000677 knockdown and exogenous CCND1 restoration on the clone formation capability of NSCLC cells NCI-H1299.

## Discussion

Recent years, circRNAs, as a newly identified noncoding RNA, have become an intense focus of research. In the present study, we reported overexpressed circ_0000677 in NSCLC patients and examined the relationship between relative circ_0000677 expression and clinical prognosis of NSCLC patients. Functionally, we found circ_0000677 could drive NSCLC cell proliferation and knockdown of circ_0000677 led to an attenuated tumorigenic capacity in BALB/c nude mice. In mechanism, circ_0000677 acted as a sponge of miR-106b and further drove miR-106b/CCND1 signaling. Taken together, these findings suggest a role for circ_0000677/miR-106b/CCND1 regulation axis in promoting NSCLC growth and progression.

CircRNAs have long been traditionally considered as abnormal products of RNA splicing. Recently, there is a growing consensus that circRNAs, mostly derived from the exon of human genes, actually functioned in multiple biological processes [26]. In the fields of cancer, aberrant expression levels of specific circRNAs were frequently associated with histological and clinical tumor features [34]. In lung cancer, particularly in NSCLC, a variety of circRNAs have been indicated to play roles in tumor growth and progression [35–37]. With regard to numerous newly identified circRNAs in NSCLC, only a few have been functionally understood. In this study, we identified circ_0000677 upregulated in NSCLC patients, which presented potential to be a novel biomarker predicting overall survival. Previous studies have demonstrated that circ_0000677 promoted cell proliferation in breast cancer and osteosarcoma [22]. Here we conducted circ_0000677 knockdown in NSCLC cell line NCI-H1299 via RNAi vector transfection, and noted an elevated proliferation capacity of NSCLC cells by clone formation analysis and immunofluorescence staining of Ki67. BALB/c nude mice subcutaneously inoculated with NSCLC cells further showed reduced tumorigenic capacity after circ_0000677 knockdown. These results are in accord with previous studies, suggesting circ_0000677 as a tumor-promoting factor.

Existing researches established that circRNAs exert a variety of modes of function, of which acting as ‘miRNA sponge’ accounts for a significant part [38]. In the present study, we predicted 3′-UTR region of circ_0000677 possesses a putative binding site for miR-106b. Further results confirmed that circ_0000677 functioned as the sponge of miR-106b and showed a markedly inverse correlation between circ_0000677 and miR-106b expression both in NSCLC cells and tissues. Consistent with most studies about circRNAs, circ_0000677 similarly can function as the sponge of miR-106b to play a role in subsequent molecular regulation.

MicroRNAs, another novel identified small non-coding RNAs, have been considered to play crucial roles in the regulation of cellular processes and maintaining tumor cell properties [39]. MiR-106b, a well investigated miRNA, is a member of miR-106b-25 cluster and has been reported to participate in regulation of multiple types of cancer tumorigenesis [40–42]. Several genes have been considered to be regulated by miR-106b. It was reported that miR-106b may stimulate cancer cell growth through targeting DAB2, FUT6, MYC, and multiple mechanisms [43–45]. Here, we identified CCND1 might be targeted by miR-106b, which possesses a potential binding site. CCND1, an essential regulated factor of G1 phase of the cell cycle, whose expression affects the cell fate [46]. We further demonstrate that CCND1 was negatively regulated by circ_0000677/miR-106b axis. Upregulation of CCND1 markedly rescued the inhibition effect of circ_0000677 konckdown on NSCLC cell proliferation. Circ_0000677/miR-106b/CCND1 regulation axis was showed to exert protumorigenic properties in NSCLC.

As compared with previous NSCLC studies, we confirmed our hypotheses by multiple means, including experiments with nude mice and robust clinical data. In addition, clinical observation from NSCLC patients indicated that higher expression of circ_0000677 predicts an unfavorable prognosis, which imply that circ_0000677 could be a reasonable target for both therapeutic and diagnostic applications. However, we are aware of the limitation that additional studies in other ethnic groups with larger number of NSCLC patients are needed to corroborate the clinical relevance of these findings. Another limitation of the present study is its lack of simultaneously comparison of the diagnostic performance of circ_0000677 with other reported biomarkers in a clinical setting. We consider this as a future direction.

## Conclusion

Taken together, the present study revealed that circ_0000677 was overexpressed in NSCLC tissues and associated with poor prognosis. Circ_0000677 functioned as a sponge of miR-106b and further regulated CCND1 in NSCLC cells, which promoted proliferation of tumor cells. These results suggested circ_0000677/miR-106b/CCND1 axis might be a promising therapeutic target in NSCLC patients.

## Highlight

- Circ_0000677 expression is elevated in NSCLC tissues than that in adjacent noncancerous tissues.
- Circ_0000677 directly targets miR-106b and regulates the cell cycle regulator CCND1.
- Circ_0000677 expression can predict overall survival in patients with NSCLC.
